# Genome-wide association between DNA methylation and alternative splicing in an invertebrate

**DOI:** 10.1186/1471-2164-13-480

**Published:** 2012-09-15

**Authors:** Kevin Flores, Florian Wolschin, Jason J Corneveaux, April N Allen, Matthew J Huentelman, Gro V Amdam

**Affiliations:** 1 School of Life Sciences, Arizona State University, PO Box 874501, 85287, Tempe, AZ, USA; 2 Department of Biotechnology, Chemistry and Food Science, Norwegian University of Life Sciences, PO Box 5003, N-1432, Aas, Norway; 3 Neurogenomics Division, The Translational Genomics Research Institute, 445 N Fifth Street, 85004, Phoenix, AZ, USA

## Abstract

**Background:**

Gene bodies are the most evolutionarily conserved targets of DNA methylation in eukaryotes. However, the regulatory functions of gene body DNA methylation remain largely unknown. DNA methylation in insects appears to be primarily confined to exons. Two recent studies in *Apis mellifera* (honeybee) and *Nasonia vitripennis* (jewel wasp) analyzed transcription and DNA methylation data for one gene in each species to demonstrate that exon-specific DNA methylation may be associated with alternative splicing events. In this study we investigated the relationship between DNA methylation, alternative splicing, and cross-species gene conservation on a genome-wide scale using genome-wide transcription and DNA methylation data.

**Results:**

We generated RNA deep sequencing data (RNA-seq) to measure genome-wide mRNA expression at the exon- and gene-level. We produced a *de novo* transcriptome from this RNA-seq data and computationally predicted splice variants for the honeybee genome. We found that exons that are included in transcription are higher methylated than exons that are skipped during transcription. We detected enrichment for alternative splicing among methylated genes compared to unmethylated genes using fisher’s exact test. We performed a statistical analysis to reveal that the presence of DNA methylation or alternative splicing are both factors associated with a longer gene length and a greater number of exons in genes. In concordance with this observation, a conservation analysis using BLAST revealed that each of these factors is also associated with higher cross-species gene conservation.

**Conclusions:**

This study constitutes the first genome-wide analysis exhibiting a positive relationship between exon-level DNA methylation and mRNA expression in the honeybee. Our finding that methylated genes are enriched for alternative splicing suggests that, in invertebrates, exon-level DNA methylation may play a role in the construction of splice variants by positively influencing exon inclusion during transcription. The results from our cross-species homology analysis suggest that DNA methylation and alternative splicing are genetic mechanisms whose utilization could contribute to a longer gene length and a slower rate of gene evolution.

## Background

DNA methylation, specifically the methylation of cytosine’s within CpG (Cytosine-phosphate-Guanine) dinucleotides, is an epigenetic signature used for the regulation of gene expression in eukaryotes [1]. Recently, the genome-wide location of DNA methylation was determined in 20 eukaryotic species [2-6]. It was revealed that the functional targets of DNA methylation vary across species with intragenic DNA methylation (inside gene bodies) being generally more conserved than promoter methylation [5,7-15].

Promoter methylation is a well-characterized mark used for gene silencing in vertebrates [16,17]. In contrast, it has been difficult to infer a conserved regulatory function for intragenic DNA methylation because the relationship between intragenic DNA methylation and transcription varies across species [5]. While it is known that intragenic DNA methylation frequently occurs within actively transcribed genes [7,8,11-13], its biological functions remain elusive and may vary depending on the organism and the exact nature of the DNA methylation patterns.

A positive linear correlation between intragenic DNA methylation and gene expression has been demonstrated in the anemone (*Nematostella vectensis*) and the silkworm (*Bombyx mori*) [4,5]. In other eukaryotes, including the honeybee, a parabolic relationship was reported in which the majority of moderately transcribed genes are more methylated than lowly or highly expressed genes, implying a lack of a positive correlation between DNA methylation and transcription at the gene level in these species [5]. In concordance with this observation, it has recently been proposed that intragenic DNA methylation, and more precisely exon DNA methylation, may instead be associated with alternative splicing events [18,19]. However, it remains to be determined if we can observe such a relationship on a genome-wide scale. In addition, it has been reported that gene body methylation in *Arabidopsis thaliana* and honeybees is positively associated with gene length and gene conservation [20,21], and that gene length between honeybee and fruit fly are highly correlated [20]. These associations bear the question whether gene length, the methylation status of genes, and/or the propensity for splicing are indicators of evolutionary sequence conservation.

The location of DNA methylation in the honeybee genome portends a genome-wide relationship between exon DNA methylation and transcription. Approximately ~80% of CpG methylation in the honeybee genome is specifically confined to the exons in about half of all genes [18]. In comparison, CpG methylation in plants and humans is spread out over several functional targets such as introns, exons, promoters, repeats, and transposons [6,14]. We sought to test (1) if there is an overall correlation between DNA methylation and gene expression patterns in honeybees; (2) whether DNA methylation in exons correlates with the inclusion of exons in transcription on a genome-wide scale; (3) if DNA methylation is generally associated with the construction of splice variants in the honeybee; and (4) if gene length, the presence of DNA methylation and/or alternative splicing in honeybee genes is associated with higher cross-species evolutionary conservation.

## Results and discussion

In order to investigate the relationship between DNA methylation and transcription across the entire honeybee genome, we generated genome-wide transcription data using RNA-seq (pooled RNA from 20 individuals) and analyzed them in conjunction with available genome-wide DNA methylation data from honeybees (pooled DNA from 50 individuals) [18]. Both data sets were aligned to the honeybee Amel 2.0 genome. The bees from the two datasets were of the same caste, sex (female workers), and tissue (brain).

### The relationship between DNA methylation and gene expression

We tested whether honeybee DNA methylation is related to expression at the level of whole genes using both a linear (Pearson’s) and non-linear (Spearman’s) correlation analysis. We restricted this analysis to genes that are both expressed and methylated because these genes are the most biologically relevant for our hypothesis and data points for unexpressed or non-methylated genes could only weaken the correlation. Even with this restriction, we did not find a significant genome-wide positive correlation between gene DNA methylation and gene expression (Pearson’s, N = 619, P = .2175, r = .0496; Spearman’s, N = 619, P = .055, rho = .0772). The relative methylation value (mCG/CG) was used to calculate gene DNA methylation intensity using data from the entire gene body, including introns. Similar non-significant results were found using the absolute methylation value (mCG/length), another standard calculation of DNA methylation intensity from BS-seq data (Additional file 1: Figure S1).

These results show that intragenic DNA methylation in the honeybee brain is unlikely to act in the suppression of gene transcription on a genome-wide scale. They are consistent with previous findings by Zemach and colleagues who investigated the relationship between DNA methylation and gene expression on a whole-organism level (Additional file 1: Figure S1 for comparison to the data from Zemach and colleagues) [5].

### The relationship between DNA methylation and exon expression

**Figure 1:**
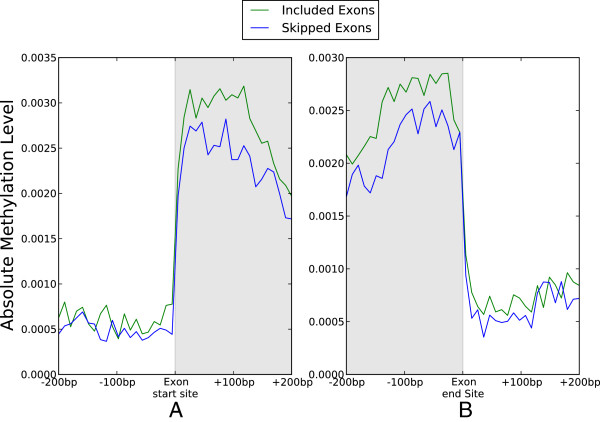
**The distribution of DNA methylation among included exons vs. skipped exons.** The plots in (**A**) and (**B**) illustrate that exons that are included in transcription are higher methylated than exons that are skipped during transcription. The absolute methylation (mCG/length) around exons was calculated by dividing the regions +/− 200 bp within the start site (**A**) or end site (**B**) of either included exons or skipped exons into 20 equal intervals. Plots show the methylation level within each interval. Included exons have significantly more methylation than skipped exons in regions beginning after the exon start site and just before the exon end site (P < .05, Wilcoxon rank-sum test in each interval). Genome-wide DNA methylation data were obtained from BS-seq [18].

Given the suggested link between exon expression and DNA methylation in honeybees ( [18]) we next tested whether a positive relationship between DNA methylation and expression exists on the level of exons, instead of the whole gene. To address this topic we analyzed BS-seq and RNA-seq data among exons within genes that are both methylated and expressed, for reasons similar to those stated above. We calculated the distribution of DNA methylation across the start and end sites of exons that were either included or skipped during transcription. We found that, overall, exons included in the gene transcript contained significantly more DNA methylation than skipped exons just after the exon start site and before the exon end site (Figure 1A and 1B, Additional file 1: Figure S2A and Additional file 1: S2B). This relationship is consistent with previous predictions based on an analysis of transcription and DNA methylation data from a single gene ( [18]), and distinctly ends at intron-exon junctions (Figure 1 A and 1B). Lyko and colleagues recently showed that DNA methylation is likely to co-localize to the proximity of alternative splicing events in the honeybee by analyzing DNA methylation data for 169 genes with alternatively spliced introns [18]. To investigate this result on a genome-wide scale, we tested for an association between DNA methylation and expression among introns. Although we did not find that intron inclusion and the prevalence of DNA methylation inside introns (within 200 bp of the intron start or end site) were correlated (Additional file 1: Figure S2C and S2D), the positive relationship between exon methylation and expression led us to investigate the possibility that DNA methylation is connected to the formation of splice variants on a genome-wide scale.

### DNA methylation in the honeybee is positively associated with the occurrence of splicing events on a genome-wide scale

**Figure 2:**
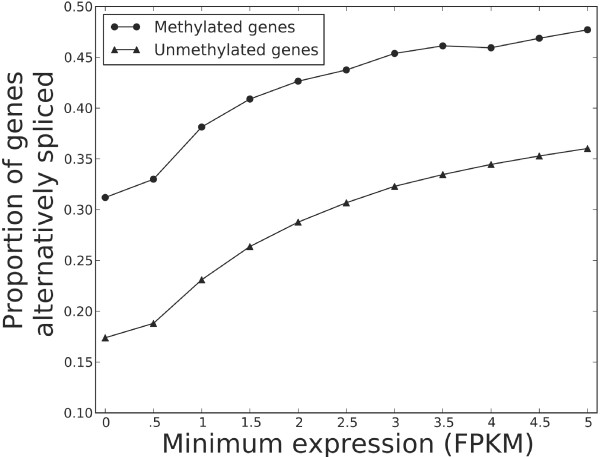
**Methylated genes are enriched for alternative splicing.** Genome-wide DNA methylation data was overlaid with genes and the occurrence of alternative transcripts predicted from a *de novo* transcriptome assembly. The proportion of methylated genes that were alternatively spliced (triangles) was significantly greater than for unmethylated genes (circles) for several minimum expression thresholds (Fisher’s exact test, P < 1e-10 for each minimum FPKM); expression is measured as the expected number of fragments per kilobase of transcript per million reads (FPKM). We tested several minimum FPKM values to show that enrichment is robust despite the possibility that transcript abundance estimation may be inaccurate at low expression (FPKM). Raw data were the same as for Figure 1.

To test whether methylated genes are enriched for splice variants when compared to unmethylated genes we assembled a honeybee transcriptome that included alternatively spliced transcripts using a *de novo* gene prediction from genome-wide RNA-seq data. We found that alternative transcripts occurred significantly more often in methylated genes as compared to unmethylated genes (Fisher’s exact test, P < 1e-10, Figure 2), over several different expression thresholds to ensure RNA-seq data quality. In contrast to our above results that suggest a general association between methylation and expression at the exon level, these data indicate that intragenic DNA methylation is positively related with alternative splicing at the gene level. This result corroborates on a genome-wide scale what has previously been predicted on the basis of individual genes: that honeybees display a close relationship between DNA methylation and alternative splicing. Given prior observations that DNA methylation and gene length appear to be associated in the honeybee, we decided to test whether DNA methylation, splicing, and gene length are linked.

### Gene length, DNA methylation, and alternative splicing are associated with the conservation of honeybee genes

**Figure 3:**
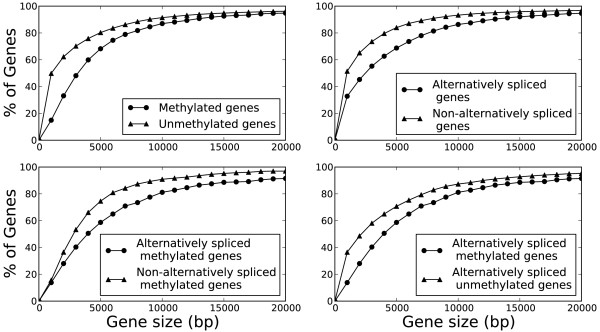
**Alternative splicing and DNA methylation in genes is associated with a longer gene length.** The cumulative distribution function (CDF) for the size of a gene (the size of the gene body, including introns, is the number of base pairs (bp) in the gene annotation) is plotted for several categories of gene methylation and alternative splicing. Top left panel: The CDFs of methylated and unmethylated genes show that methylated genes are longer than unmethylated genes. Top right panel: Alternatively spliced genes are longer than non-alternatively spliced genes. Bottom left panel: Methylation is also associated with longer gene size among non-alternatively spliced genes. Bottom right panel: Methylation is associated with longer gene size among alternatively spliced genes. Splice variants were annotated by assembling a *de novo* transcriptome from RNA-seq data. The length of a gene is defined to be the maximum length from any splice variant (including introns) of that gene. The significance of shifted distributions was quantified by using the Wilcoxon rank sum test with continuity correction (P < 2.2e-16 for each panel). Genome-wide DNA methylation data were obtained from BS-seq [18].

Our data on associations between gene length, DNA methylation, and alternative splicing show that, in the honeybee, genes that are methylated and/or alternatively spliced are longer (Figure 3) and tend to have more exons (Additional file 1: Figure S3) than genes that are not methylated and not alternatively spliced. We also verified previous observations that methylated honeybee genes are higher conserved across taxa than unmethylated genes (Table 1) [18,22]. Moreover, we found that alternatively spliced honeybee genes are higher conserved than non-alternatively spliced genes (Table 1). Finally, we observed that methylated honeybee genes that are also alternatively spliced were even higher conserved across species than methylated genes that were not alternatively spliced (Additional file 1: Table S1) or than alternatively spliced genes that are not methylated (Additional file 1: Table S2). These associations suggest that gene length, DNA methylation, and alternative splicing are positively linked to gene conservation. Compared to non-methylated and non-alternatively spliced genes, methylated and/or spliced genes can result in a greater variety of transcripts. Thus, by varying the methylation and splicing pattern, genes can assume novel functions without a necessary change in their primary sequence. This may explain why the respective genes can afford a higher conservation – they realize variation in different ways, e.g. by variation in master regulators of DNA methylation and splicing.

**Table 1:** DNA methylation or alternative splicing in honeybee genes is associated with higher cross-species evolutionary conservation

	**Proportion of methylated genes**	**Proportion of unmethylated genes**	**Methylated vs. unmethylated P-value**	**Proportion of alternatively spliced genes**	**Proportion of non-alternatively spliced genes**	**Alternatively spliced vs. Non-alternatively spliced P-value**
Conserved in *H. sapiens*	0.215	0.082	7.20E-69	0.189	0.13	8.79E-10
Conserved in *C. intestinalis*	0.142	0.053	1.86E-45	0.125	0.084	2.83E-07
Conserved in *A. pisum*	0.24	0.136	5.40E-35	0.259	0.159	1.17E-21
Conserved in *N. vitripennis*	0.426	0.278	9.26E-47	0.445	0.312	1.33E-25
Conserved in *D. melanogaster*	0.265	0.163	6.22E-31	0.3	0.179	7.23E-28

Cross-species gene conservation was determined by protein BLAST (E-value cutoff of 1E-150). A significantly higher proportion of methylated honeybee genes are conserved across several species and taxa (*Homo sapiens, Ciona intestinalis, Acryothisum pisum, Nasonia vitripennis, Drosophila melanogaster*) than unmethylated genes (Fisher’s exact test, P-values shown in column 4). Similarly, a significantly higher proportion of alternatively spliced genes are conserved than non-alternatively spliced genes (P-values in column 7). Raw data were the same as for Figure 1.

## Conclusions

These results support a genome-wide positive association between splicing events and exon methylation in the honeybee. While we cannot say whether DNA methylation causes splicing events, it is tempting to surmise that DNA methylation in exons can mark the chromatin for modifications that reduce the chance of exon-skipping, leading to a higher rate of exon inclusion during the process of transcription. Interestingly, our data also show that skipped exons are methylated at lower levels than expressed exons, but also contain higher levels of DNA methylation than surrounding introns (Figure 1). It remains to be determined whether or not there is a possible role for this DNA methylation within skipped exons. One possibility that was recently uncovered by Shukla and colleagues in mammals is that exon-specific DNA methylation may affect exon-skipping by interfering with CCCTC-binding factor (CTCF) binding to the DNA. CTCF binding can promote the inclusion of nearby exons by causing RNA polymerase II pausing, and thus interference with CTCF binding has a reciprocal effect on exon inclusion [23]. These observations support the hypothesis that the effect of DNA methylation, i.e. either causing a repressed (heterochromatin) or open (euchromatin) chromatin state, can vary depending on the species, sequence context, DNA binding proteins, and the histone modifying enzymes that are associated with methylated DNA [16,24].

The finding that DNA methylation in honeybees is primarily targeted to exons ( [18]) and the genome-wide positive association between exon expression and DNA methylation that we report here suggests that honeybees may be a compelling organism in which to establish a functional link between DNA methylation and other epigenetic mechanisms, such as histone modifications. Global correlations between intragenic DNA methylation and specific histone modifications have been difficult to establish in other species in part because DNA methylation has many functional genomic targets (e.g. introns, exons, promoters, repeats, etc.) and therefore may play more diverse roles than in honeybees [25]. For example, Hahn and colleagues recently analyzed DNA methylation in human cell lines and demonstrated that although intragenic DNA methylation co-localizes with H3K9 methylation (a modification usually associated with DNA methylation in plant and mammalian heterochromatin repeats) and H3K36 trimethylation (a modification associated with DNA hyper methylation and transcriptional elongation, i.e. euchromatin), the co-localization of these marks within gene bodies is largely independent [26]. Because honeybees have a relatively simple DNA methylation landscape, examining the link between DNA methylation and histone modifications within honeybee exons, for example by analyzing genome-wide BS-seq data together with ChIP-seq data for histone modifications, may yield different results than in humans.

Overall, the honeybee methylome exemplifies that DNA methylation can be targeted with a resolution that differentiates intron-exon boundaries. This capacity for exon DNA methylation targeting appears to be evolutionarily conserved in humans [27]. Thus, the use of exon methylation to control splice variants genome-wide in a context-specific manner may be a basal function for both vertebrate and invertebrate DNA methylation systems. Future studies should perform manipulative experiments to causally test the predicted link between DNA methylation and gene splicing. For example, knocking down or de-activating the *de novo* DNA methyltransferase (DNMT3), shown to be feasible in the honeybee by Kucharski and colleagues [28], should change the splice variant pool during life-stages when DNA methylation marks are established or changed.

Biological roles of exon DNA methylation may differ or coincide between species and could involve basic biological processes, organismal and behavioral plasticity, as well as tissue differentiation. Elango and colleagues used a computational prediction to show that methylation is likely to occur in genes associated with metabolism and housekeeping functions of gene transcription and translation [29]. Lyko and colleagues used BS-seq and found that brain DNA methylation patterns differ between queens and workers, suggesting that DNA methylation is involved in the regulation of caste-specific genes that may influence behavior in honeybees [18]. Our results demonstrate that one way DNA methylation could regulate these genes is by altering the splice variant landscape. In future studies, the correlation between DNA methylation and exon expression that we demonstrated for the honeybee could become stronger by using DNA and RNA extracted from individuals matched for age and behavior. By using such stringency in experimental design, it will likewise be beneficial to study the associations between DNA methylation and splice variant formation across tissues during development, maturation, and aging in a variety of insect species with different life-history dynamics and behaviors. This may include species with caste polymorphisms (e.g. honeybee, ants) as well as solitary species with different lifestyles (e.g. the parasitic jewel wasp and the silkmoth). From a mechanistic point of view it will be especially instructive to compare species with a positive correlation between DNA methylation and gene expression (e.g. the silkworm) to the ones with a negative or no correlation between DNA methylation and gene expression (e.g. the honeybee). The knowledge gained in insects can then be tested for its evolutionary conservation in organisms with a more complex DNA methylation landscape. By using RNA-seq to predict splice variants in various honeybee tissues we also can enhance our understanding of the associations between gene DNA methylation, cross-species conservation, length, and expression breadth across tissues that have recently been established in the honeybee [20,22,30]. For example, if intragenic DNA methylation mediates the generation of many splice variants from the same gene, this may enable a methylated gene to take on new functions without perturbing the existing isoforms. This mechanism would benefit methylated honeybee genes, which tend to be highly conserved across species [18,25]. We also speculate that genes that are longer and contain more exons could be preferentially targeted for intragenic DNA methylation due to their larger number of possibilities for alternative splicing. Moroever, the methylation of a gene may enable its broad expression in several tissues by influencing the expression of tissue-specific splice variants that have tissue-specific functions.

## Methods

Honeybees. Wildtype bees (*Apis mellifera carnica*) were raised at Arizona State University (Tempe, USA). Returning from foraging flights with pollen loads individuals were collected at the hive entrance and put into ice-cold 70% ethanol.

Dissections and RNA extraction. The brains of 20 individuals were dissected on ice and RNA was isolated using the TRIzol® Plus RNA isolation kit from Invitrogen (Carlsbad, CA, USA), following the manufacturers instructions.

DNA methylation data. Genome-wide DNA methylation data from bisulfite sequencing (BS-seq) of honeybee worker brains were obtained from Lyko *et al.*[18]. The BS-seq data we analyzed were generated from reads mapped to the Amel_2.0 genome and bases assessed for methylation as previously described by Lyko *et al.*[18]. The methylation intensity, mCG, was calculated at each CpG location as (# methylated reads)/(# methylated reads + # unmethylated reads). Genes that contained at least one methylated CpG with mCG > 0 were classified as methylated.

Generation of RNA-seq data. RNA was purified, DNAse treated, and eluted with 30 ul of RNAse free H20, using the RNeasy Mini Kit and the RNase-Free DNase Set (Qiagen, Valencia, CA, USA). 50 ng of pooled RNA was reverse transcribed with oligo-d(T) and random primers into cDNA, and linearly amplified using NuGEN’s Ovation RNA-Seq protocol (NuGEN, San Carlos, CA, USA). Samples were then fragmented (Covaris, Woburn, MA, USA), end-repaired, d-A tailed, and adaptor-ligated with the NEBNext DNA Sample Prep Reagent set (New England Biolabs [NEB], Ipswich, MA, USA). The ligation product was then run on a 2% TAE agarose gel to excise an approximately 300 bp fragment. Enrichment PCR (15 cycles) was performed using Phusion High-Fidelity PCR Master Mix (NEB). Libraries were then quantified on a bioanalyzer with a High Sensitivity DNA chip (Agilent Technologies, Santa Clara, CA, USA). Whole transcriptome paired-end data was generated with the HiSeq 2000 (Illumina, San Diego, CA). The sequences have been submitted to the National Center for Biotechnology Information Sequence Read Archive database under accession no. SRA051462. 116,791,866 total reads of length 104 bases were generated passing default Illumina thresholds. 14 bases were clipped off the end of the second read to remove low-quality overlapping ends. Reads were aligned to Amel_2.0 with TopHat [31] (version 1.2.0, bowtie version 0.12.7) using default parameters. In total, 64,321,565 reads were mapped and we identified 96,823 exon junctions. We used the “markduplicates” command in Picard (version 1.64) and found 25,956,078 duplicate reads (40.35%) in our data [32]. In accordance with typical RNA-seq data analysis approaches [32,33], the duplicate reads were retained for accurate transcript quantitation.

Calculation of exon and gene expression. Gene expression for Figure 3 and Additional file 1: Figure S1 is measured as the expected number of fragments per kilobase of transcript per million reads (FPKM), which quantifies the expression of an entire gene based on the expected number of read fragments that are located within coding regions [33]. Currently, there is no method to calculate the FPKM for individual exons, so instead we calculated the number of reads per kilobase of transcript per million reads (RPKM), a standard quantification of expression for RNA-seq data [34], for each exon as a measure for exon-level expression for Figure 1 and Additional file 1: Figures 2A and 2B. Exons with RPKM > 0 were classified as exons included in gene transcription, whereas exons with RPKM = 0 were classified as exons that were skipped during gene transcription. Introns were similarly classified for Additional file 1: Figures 2C and 2D. Alternative transcripts were derived from a *de novo* assembly of the honeybee transcriptome (a total of 72,479 unique transcripts) from RNA-seq data using Cufflinks (version 0.9.3) [33]. The FPKM is simultaneously calculated when alternative transcripts are predicted with Cufflinks, thus the FPKM was used as a measure of expression for alternative transcripts. Statistical analysis was done in the R programming language (version 2.10.1) and Python (version 2.6.4).

Calculation of cross species gene conservation. Conservation of *Apis mellifera* genes within other species was determined by BLAST (Basic Local Alignment Search Tool, version 2.2.18), specifically the blastp tool. All protein annotations (including those corresponding to Amel_2.0) were downloaded from the NCBI ftp site ( http://www.ncbi.nlm.nih.gov/Ftp/). Genes were considered conserved if BLAST resulted in an E-value of less than 1e-150 [19,22]. For the analysis in Table 1 and Additional file 1: Tables S1 and S2, splice variants from the *de novo* transcriptome were aligned with gene annotations from Amel_2.0 using nucleotide BLAST (with E-value cutoff of 1e-50) to determine which genes are alternatively spliced.

## Competing interests

The authors declare that they have no competing interests.

## Authors’ contributions

KF and FW wrote the manuscript, GVA revised the manuscript, JJC and ANA generated the RNA-seq data, KF performed the statistical analyses, MJH supervised the collection of the RNA-seq data. All authors read and approved the final manuscript.

## Data deposition

The sequencing data reported in this paper have been deposited in the Sequence Read Archive database (accession no. SRA051462).

## Supplementary Material

Additional file 1 **Figure S1. **We did not find an overall correlation between expression and DNA methylation within gene bodies in the honeybee brain. For comparison to other published results, the relative methylation level (mCG/CG, top panel) and absolute methylation (mCpG/length, bottom panel) were calculated for 100bp intervals plotted within gene-expression quintiles. We included data from the regions 2kbp up/down stream of the translation start site (left-most zero on the x-axis) and 2kbp up/downstream of the translation end site (right-mist zero on the x-axis), including introns. The 5^th^ expression quintile is the highest expressed. Plots show the methylation level within each interval. DNA methylation data were obtained from BS-seq ( [1]) and expression data were based on RNA-seq. These data do not show the parabolic relationship between DNA methylation and whole gene transcription demonstrated by Zemach and colleagues [2]. This discrepancy may be related to the fact that we did not use the same tissue as Zemach and colleagues (brains vs. whole bodies). Figure S2. Exons included in transcription are more methylated than skipped exons and introns included in transcription have the same level of methylation as skipped introns. The plots in (A) and (B) illustrate that DNA methylation in exons included in gene transcription is significantly higher than DNA methylation in skipped exons. The absolute methylation (mCG/length) around exons was calculated by dividing the regions +/- 200bp within the start site (A) or end site (B) of either included exons or skipped exons into 20 equal intervals. These calculations yielded a distribution of methylation values for included exons and for skipped exons within each interval. The Wilcoxon rank-sum test with continuity correction was used within each interval to determine whether the included exons had significantly more DNA methylation than skipped exons. Significant data points are plotted in red (P-value < .05) and non-significant data points are plotted in black (P-value > .05). The dashed lines, when shown, are P-value = .05 (equivalent to -log(P-value) = 1.3). Similar calculations were performed for included vs. skipped introns around the intron start site (C) or end site (D). Included introns are methylated at the same level as skipped introns. However, the inclusion of introns in transcription does correlate with higher levels of DNA methylation at approximately 100bp into the downstream exon. DNA methylation data were obtained from BS-seq [1]. Figure S3. Alternative splicing and DNA methylation in genes is associated with a greater number of exons. The cumulative distribution function (CDF) for the number of exons in a gene is plotted for several categories of gene methylation and alternative splicing. Top left panel: The CDFs of methylated and unmethylated genes show that methylated genes contain more exons than unmethylated genes. Top right panel: Alternatively spliced genes contain more exons than non-alternatively spliced genes. Bottom right panel: Methylation is also associated with a higher number of exons among alternatively spliced genes. Bottom left panel: Alternative splicing is also associated with a higher number of exons among methylated genes. Splice variants were annotated by assembling a *de novo* transcriptome from RNA-seq data. The number of exons in an alternatively spliced gene was defined to be the maximum number of exons from any splice variant of that gene. The significance of shifted distributions was quantified by using the Wilcoxon rank sum test with continuity correction (P < 2.2e-16 for each panel). DNA methylation data were obtained from BS-seq [1]. Table S1. Methylated honeybee genes that are also alternatively spliced are more evolutionarily conserved than methylated genes that are not alternatively spliced. Cross-species gene conservation was determined by protein BLAST (E-value cutoff of 1e-150). Here, the evolutionary conservation analysis is restricted to the data for methylated honeybee genes. There is a significantly higher proportion of methylated honeybee genes that are also alternatively spliced that are conserved across several species (*Homo sapiens, Ciona intestinalis, Acrythosiphon pisum, Nasonia vitripennis, Drosophila melanogaster*) as compared to methylated genes that are not alternatively spliced (Fisher’s exact test, P-values shown in column 4). DNA methylation data were obtained from BS-seq [1]. Table S2. Alternatively spliced honeybee genes that are also methylated are more evolutionarily conserved than alternatively spliced genes that are not methylated. Cross-species gene conservation was determined by protein BLAST (E-value cutoff of 1e-150). Here, the evolutionary conservation analysis is restricted to the data for alternatively spliced honeybee genes. There is a significantly higher proportion of alternatively spliced honeybee genes that are also methylated that are conserved across several species (*Homo sapiens, Ciona intestinalis, Acrythosiphon pisum, Nasonia vitripennis, Drosophila melanogaster*) as compared to alternatively spliced genes that are not methylated (unmethylated) (Fisher’s exact test, P-values shown in column 4). DNA methylation data were obtained from BS-seq [1]. Click here for file
