# Supplementary material for: Genome-wide association between DNA methylation and alternative splicing in an invertebrate
Source: BMC Genomics. 2012 Sep 15;13:480. doi: 10.1186/1471-2164-13-480 (PMC3526459; doi:10.1186/1471-2164-13-480)
Supplement: Additional file 1 — Figure S1. We did not find an overall correlation between expression and DNA methylation within gene bodies in the honeybee brain. For comparison to other published results, the relative methylation level (mCG/CG, top panel) and absolute methylation (mCpG/length, bottom panel) were calculated for 100bp intervals plotted within gene-expression quintiles. We included data from the regions 2kbp up/down stream of the translation start site (left-most zero on the x-axis) and 2kbp up/downstream of the translation end site (right-mist zero on the x-axis), including introns. The 5th expression quintile is the highest expressed. Plots show the methylation level within each interval. DNA methylation data were obtained from BS-seq ( [1]) and expression data were based on RNA-seq. These data do not show the parabolic relationship between DNA methylation and whole gene transcription demonstrated by Zemach and colleagues [2]. This discrepancy may be related to the fact that we did not use the same tissue as Zemach and colleagues (brains vs. whole bodies). Figure S2. Exons included in transcription are more methylated than skipped exons and introns included in transcription have the same level of methylation as skipped introns. The plots in (A) and (B) illustrate that DNA methylation in exons included in gene transcription is significantly higher than DNA methylation in skipped exons. The absolute methylation (mCG/length) around exons was calculated by dividing the regions +/- 200bp within the start site (A) or end site (B) of either included exons or skipped exons into 20 equal intervals. These calculations yielded a distribution of methylation values for included exons and for skipped exons within each interval. The Wilcoxon rank-sum test with continuity correction was used within each interval to determine whether the included exons had significantly more DNA methylation than skipped exons. Significant data points are plotted in red (P-value < .05) and non-significant data poin [file 1471-2164-13-480-S1.doc]

**Supplementary Material**

Supplementary figure S1. **We did not find an overall correlation between expression and DNA methylation within gene bodies in the honeybee brain.** For comparison to other published results, the relative methylation level (mCG/CG, top panel) and absolute methylation (mCpG/length, bottom panel) were calculated for 100bp intervals plotted within gene-expression quintiles. We included data from the regions 2kbp up/down stream of the translation start site (left-most zero on the x-axis) and 2kbp up/downstream of the translation end site (right-mist zero on the x-axis), including introns. The 5^th^ expression quintile is the highest expressed. Plots show the methylation level within each interval. DNA methylation data were obtained from BS-seq ([1]) and expression data were based on RNA-seq. These data do not show the parabolic relationship between DNA methylation and whole gene transcription demonstrated by Zemach and colleagues [2]. This discrepancy may be related to the fact that we did not use the same tissue as Zemach and colleagues (brains vs. whole bodies).


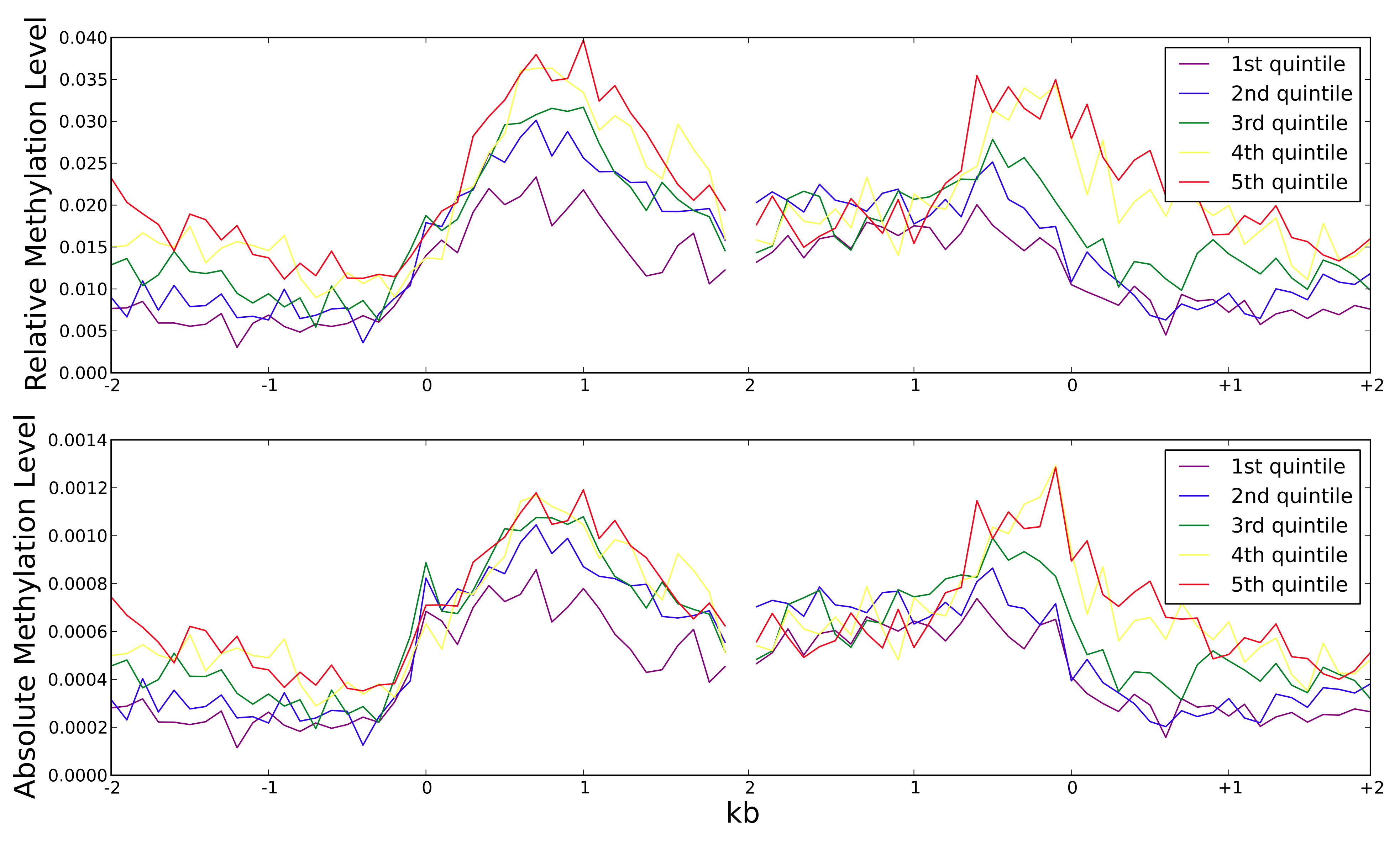


Supplementary figure S2. **Exons included in transcription are more methylated than skipped exons and introns included in transcription have the same level of methylation as skipped introns.** The plots in (A) and (B) illustrate that DNA methylation in exons included in gene transcription is significantly higher than DNA methylation in skipped exons. The absolute methylation (mCG/length) around exons was calculated by dividing the regions +/- 200bp within the start site (A) or end site (B) of either included exons or skipped exons into 20 equal intervals. These calculations yielded a distribution of methylation values for included exons and for skipped exons within each interval. The Wilcoxon rank-sum test with continuity correction was used within each interval to determine whether the included exons had significantly more DNA methylation than skipped exons. Significant data points are plotted in red (P-value < .05) and non-significant data points are plotted in black (P-value > .05). The dashed lines, when shown, are P-value = .05 (equivalent to -log(P-value) = 1.3). Similar calculations were performed for included vs. skipped introns around the intron start site (C) or end site (D). Included introns are methylated at the same level as skipped introns. However, the inclusion of introns in transcription does correlate with higher levels of DNA methylation at approximately 100bp into the downstream exon. DNA methylation data were obtained from BS-seq [1].


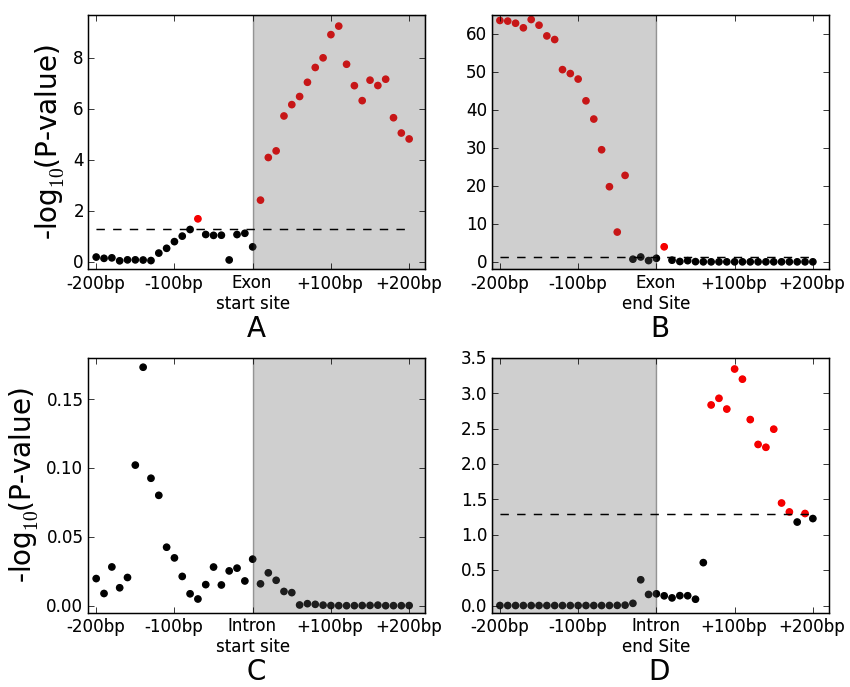


Supplementary figure S3. **Alternative splicing and DNA methylation in genes is associated with a greater number of exons.** The cumulative distribution function (CDF) for the number of exons in a gene is plotted for several categories of gene methylation and alternative splicing. Top left panel: The CDFs of methylated and unmethylated genes show that methylated genes contain more exons than unmethylated genes. Top right panel: Alternatively spliced genes contain more exons than non-alternatively spliced genes. Bottom right panel: Methylation is also associated with a higher number of exons among alternatively spliced genes. Bottom left panel: Alternative splicing is also associated with a higher number of exons among methylated genes. Splice variants were annotated by assembling a *de novo* transcriptome from RNA-seq data. The number of exons in an alternatively spliced gene was defined to be the maximum number of exons from any splice variant of that gene. The significance of shifted distributions was quantified by using the Wilcoxon rank sum test with continuity correction (P < 2.2e-16 for each panel). DNA methylation data were obtained from BS-seq [1].


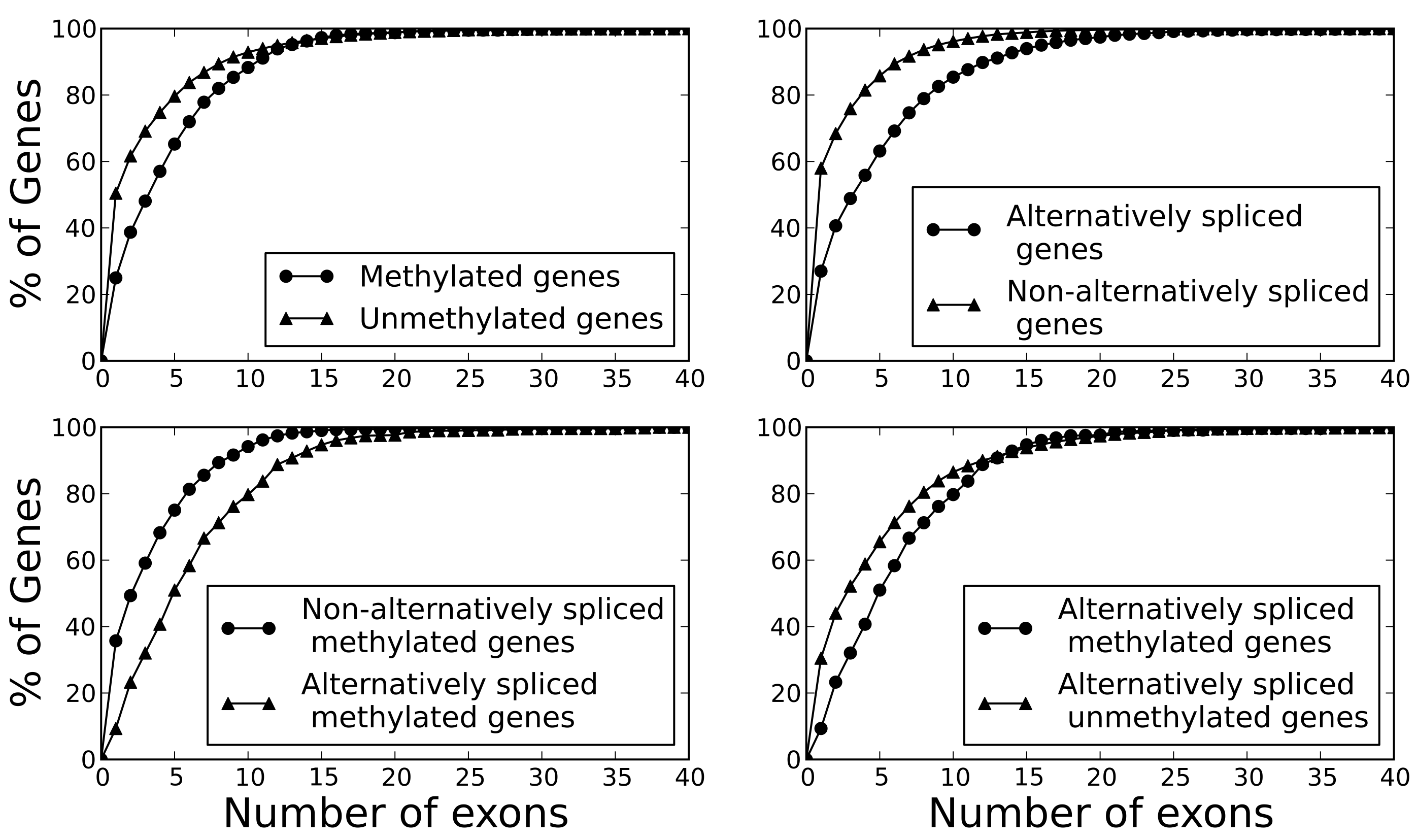


Supplementary table S1. **Methylated honeybee genes that are also alternatively spliced are more evolutionarily conserved than methylated genes that are not alternatively spliced.** Cross-species gene conservation was determined by protein BLAST (E-value cutoff of 1e-150). Here, the evolutionary conservation analysis is restricted to the data for methylated honeybee genes. There is a significantly higher proportion of methylated honeybee genes that are also alternatively spliced that are conserved across several species (*Homo sapiens, Ciona intestinalis, Acrythosiphon pisum, Nasonia vitripennis, Drosophila melanogaster*) as compared to methylated genes that are not alternatively spliced (Fisher’s exact test, P-values shown in column 4). DNA methylation data were obtained from BS-seq [1].

| (Methylated genes only) | Proportion of alternatively spliced methylated genes | Proportion of non-alternatively spliced methylated genes | Alternatively spliced vs. Non-alternatively spliced P-value |
| --- | --- | --- | --- |
| Conserved in *H. sapiens* | 0.276 | 0.201 | 3.88E-06 |
| Conserved in *C. intestinalis* | 0.199 | 0.13 | 7.73E-07 |
| Conserved in *A. pisum* | 0.331 | 0.219 | 4.56E-11 |
| Conserved in *N. vitripennis* | 0.503 | 0.409 | 1.03E-06 |
| Conserved in *D. melanogaster* | 0.513 | 0.417 | 5.95E-07 |

Supplementary table S2. **Alternatively spliced honeybee genes that are also methylated are more evolutionarily conserved than alternatively spliced genes that are not methylated.** Cross-species gene conservation was determined by protein BLAST (E-value cutoff of 1e-150). Here, the evolutionary conservation analysis is restricted to the data for alternatively spliced honeybee genes. There is a significantly higher proportion of alternatively spliced honeybee genes that are also methylated that are conserved across several species (*Homo sapiens, Ciona intestinalis, Acrythosiphon pisum, Nasonia vitripennis, Drosophila melanogaster*) as compared to alternatively spliced genes that are not methylated (unmethylated) (Fisher’s exact test, P-values shown in column 4). DNA methylation data were obtained from BS-seq [1].

| Alternatively spliced genes only | Proportion of alternatively spliced genes methylated | Proportion of alternatively spliced genes unmethylated | Methylated vs. Unmethylated  P-value |
| --- | --- | --- | --- |
| Conserved in  *H. sapiens* | 0.276 | 0.116 | 6.01E-18 |
| Conserved in  *C. intestinalis* | 0.199 | 0.064 | 5.90E-18 |
| Conserved in  *A. pisum* | 0.331 | 0.199 | 2.99E-10 |
| Conserved in  *N. vitripennis* | 0.503 | 0.397 | 7.29E-06 |
| Conserved in  *D. melanogaster* | 0.513 | 0.25 | 1.66E-30 |

**Supplementary References**

1. Lyko F, Foret S, Kucharski R, Wolf S, Falckenhayn C, Maleszka R: The honey bee epigenomes: differential methylation of brain DNA in queens and workers. *PLoS Biol* 2010, 8:e1000506.

2. Zemach A, McDaniel IE, Silva P, Zilberman D: Genome-wide evolutionary analysis of eukaryotic DNA methylation. *Science* 2010, 328:916-919.
